# Oncology pharmacy units: a safety policy for handling hazardous drugs and related waste in low- and middle-income African countries—Angolan experience

**DOI:** 10.3332/ecancer.2015.575

**Published:** 2015-10-01

**Authors:** Ana Vaz da Conceição, Dora Bernardo, Lygia Vieira Lopes, Fernando Miguel, Fernanda Bessa, Fernando Monteiro, Cristina Santos, Blasques Oliveira, Lúcio Lara Santos

**Affiliations:** 1 Cancer Unit of Girassol Clinic, Comandante Gika 225, Luanda, Angola; 2 Pharmacy Department of Portuguese Institute of Oncology, Rua Dr António Bernardino de Almeida – 4200–072, Porto, Portugal; 3 Cancer Unit of Sagrada Esperança Clinic, Av Murtala Mohammed, Luanda, Angola; 4 Angolan Institute of Cancer Control, Rua Amilcar Cabral, Luanda, Angola; 5 Nurse Department of Portuguese Institute of Oncology, Rua Dr António Bernardino de Almeida – 4200–072, Porto, Portugal; 6 ONCOCIR - Education and Care in Oncology, Marçal, Luanda, Angola; 7 Experimental Pathology and Therapeutics Group and Surgical Oncology Department, Portuguese Institute of Oncology, Rua Dr António Bernardino de Almeida – 4200–072, Porto, Portugal; * These authors equally contributed to this study.

**Keywords:** Angola, chemotherapy, hazardous drugs, low- and middle-income industries, oncology pharmacy units

## Abstract

In African countries, higher rates of late-stage cancers at the time of first diagnosis are a reality. In this context, hazardous drugs (HDs), such as chemotherapy, play an important role and have immense benefits for patients’ treatment.

HDs should be handled under specific conditions. At least a class 5 environment primary engineering control (PEC), physically located in an appropriate buffer area, is mandatory for sterile HDs compounding, as well as administrative control, personal protective equipment, work practices and other engineering and environmental controls, in order to protect the environment, patient, and worker.

The aim of this study is to describe the Angolan experience regarding the development of oncology pharmacy units and discuss international evidence-based guidelines on handling HDs and related waste. Measures to incorporate modern and economical solutions to upgrade or build adequate and safe facilities and staff training, in order to comply with international guidelines in this area, are crucial tasks for African countries of low and middle income.

## Background

In 2012, low- and middle-income countries contributed 57% and 65% to the global cancer incidence and mortality, respectively [1]. These countries, already facing a heavy burden of infectious diseases, are increasingly affected by new epidemics of non-communicable diseases. Of these, cancer in particular could severely upset government and personal health care budgets [2]. The most common cancers in sub-Saharan Africa (Kaposi’s sarcoma, cervical cancer, breast cancer, and certain types of non-Hodgkin’s lymphoma) are amenable to prevention, early detection and treatment. In African countries, cancer control programmes and provision of early detection and treatment services are still under development due to this increased burden [3].

High rates of late-stage cancers at the time of first diagnosis have been well documented in low- and middle-income countries such as Angola [4].

In this context, chemotherapy plays an important role and can have immense benefits for patients when used in proper regimens with minimised risks.

It is also crucial that the receipt, storage, compound, dispense, transport, administration, cleaning and disposal of hazardous drugs (HDs), chemotherapy included, should always be performed under conditions that ensure safety of the health professionals, patients, and environment.

## Why do we need a safety policy?

Drugs considered hazardous include those that exhibit one or more of the following six characteristics for humans or animals: carcinogenicity, teratogenicity or other forms of developmental toxicity, reproductive toxicity, organ toxicity at low doses, genotoxicity and new drugs that mimic those determined hazardous [5]. Therefore, these drugs pose a potential risk to health care workers who may be exposed when handling them.

Falck published, in 1979, the first convincing evidence, in a small but controlled study, that mutagenic activity was found in the urine of patients who received chemotherapy as well as in the nurses who administered it. Since then, evidence of significant risk by occupational exposure has been published [6–8]. The main routes of cytotoxic drug exposure occur through skin contact and absorption, inhalation of aerosols and drug particles, ingestion and sharps injuries [9, 10]. In the 1980s, the USA Occupational Safety and Health Administration (OSHA) became aware of the facilities’ chemotherapy preparation practices [11].

The subsequent investigation resulted in the facilities being cited for failure providing protection for the pharmacists [12]. The safe handling programme, then implemented, was described in the American Journal of Hospital Pharmacy and became the basis for the first American Society of Hospital Pharmacists (ASHP) Technical Assistance Bulletin on Handling Cytotoxic Drugs [13, 14, 15].

Polovich wrote, in 2004, an important article explaining the risks associated with the handling of HDs and describes in detail how to minimise them [16]. Environmental contamination with hazardous drugs is also well documented and was the main reason for implementing guidelines in the construction of pharmacy facilities where cytotoxic drugs are handled [17].

Several articles identified surface contamination by chemotherapy drugs in the pharmacies and patient care areas [18, 19, 20].

## Barriers against safety policy—handling hazardous drugs

Although the benefits for the implementation of safe policies on handling hazardous drugs are well documented, several difficulties limit their application. Normally, the barriers are related to various issues, such as personnel’s knowledge, attitudes, supervisors encouraging precaution in usage, compliance, logistical and area limitations in most institutions, as well as prohibitive cost. However, American, European, and Australian guidelines agree that safety precautions for personnel and environment exposure are necessary [5, 10, 17, 21, 22, 23, 24].

## Challenges in Africa

Concerning Africa, the limitations stated previously are increased by other difficulties regarding these policies on handling HDs and waste, such as scarce infrastructures and supply chains, which cause impact in patient access, lack of healthcare providers and trained staff. These limitations should be taken into account when designing oncology pharmacy units.

Therefore, the need for appropriate facilities is essential considering that hazardous drug preparation presents a twofold challenge, that is, to limit microbial contamination to protect the patient and to limit environmental contamination by hazardous drugs, avoiding worker exposure and ensuring environmental protection. When setting up a sterile preparation room, particularly regarding pressure gradients, which protect both product and worker, maintaining acceptable and manageable costs and equity to patient access to treatment with chemotherapy is even a greater challenge. On the other hand, only chemotherapy-trained personnel will prepare and dispense cancer chemotherapy, while adequate and waste-related management would be ensured [25].

The aim of this study is to evaluate the Angolan experience regarding the development of these units, their history, difficulties, options, costs and staff training, in order to translate this knowledge into African recommendations.

## Methods

This research was conducted between January 2012 and December 2014 in Angola and started with a preliminary self-assessment based on a survey (The Cancer Units Assessment Checklist for low or middle income African countries), addressed to three hospitals in Luanda, followed by a peer review visit by auditors, in order to perform diagnosis of the situation and develop an action plan.

This survey assessed physical facilities, existence of a centralised unit, equipment for HDs preparation and infusion unit or the development plans for these, guidelines for acquisition, preparation, prescription protocols and administration of cytotoxic drugs, waste management and safety requirements [26].

**Figure 1.**
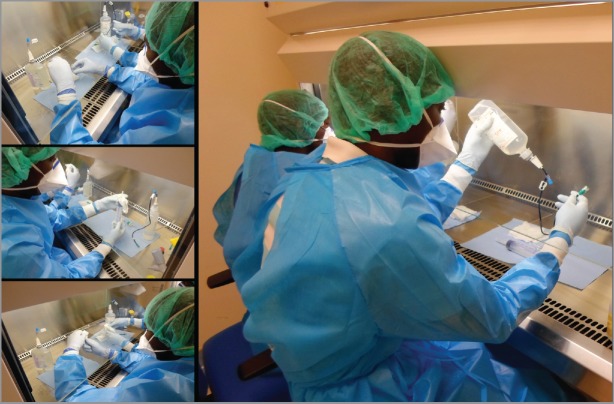
Practical training of pharmacists and pharmacy technicians. Simulated training.

The following hospitals were involved in this survey:

The Angolan Institute of Cancer Control (IACC) former National Oncology Centre in Luanda, the only specialised cancer public hospital and the main centre for the treatment of patients with patients in Angola. Each year, around a 1000 new patients are admitted and assessed, within a multidisciplinary approach. Over the past 39 years, this hospital has been the only support for patients with cancer in this country. The hospital performs around 7000 chemotherapy preparations per year (Figure 1).

Girassol Clinic (GC), a private clinic, belonging to the public company Sonangol, able to perform diagnosis, surgery, and radiotherapy. Chemotherapy is infused in a dedicated ward with cytotoxic drugs, currently being prepared at the IACC, but planning to rebuild the oncology pharmacy unit.

Sagrada Esperança Clinic (SEC), a private clinic, belonging to a public company named Endiama, performs cancer diagnosis and surgery. Chemotherapy treatments are prepared and infused at the IACC. There are plans for the construction of an oncology pharmacy.

Auditors evaluated whether the organisations met the quality standards according to the assessment checklist as shown in Table 1.

Locally, the auditors identified the gaps between pharmacy procedures, inadequacies in the facilities and then developed an action plan for USP 797 chapter compliance. They also addressed changes needed in the facilities, equipment and standard operating procedures. The workload demands for compounding on site were also evaluated. The development of each assigned oncology pharmacy and their history, difficulties, options, costs, and staff training were recorded and studied.

After review, the auditors delivered a report, identifying quality concerns and recommendations for an action plan for each hospital, which is resumed in Table 2.

Two years later, in 2014, a second audit took place, having the same team of auditors, taking into account the recommendations previously provided.

## Results

After the second audit rolled in, the policies for handling HDs, related wastes and units, are evolved as follows:

### Girassol Clinic

**Figure 2.**
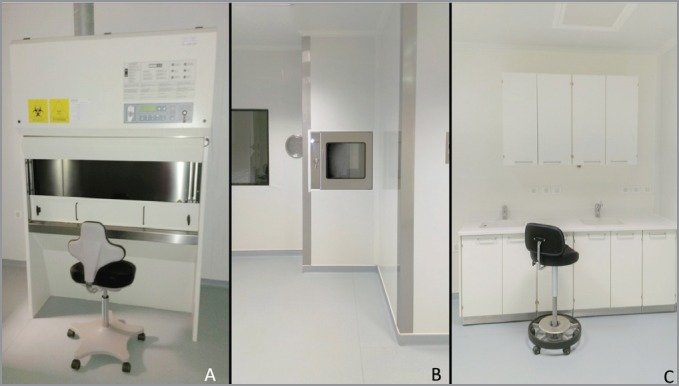
Girassol Clinic oncology pharmacy unit. (A) Primary engineering control (BSC type II) in buffer room. (B) Pass through with HEPA filters. (C) Anteroom (making ready area) meeting USP 797 criteria.

The initial building construction plan and equipment acquired for this clinic included a cancer unit (radiotherapy, nuclear medicine, oncology pharmacy and ambulatory infusion areas, operating theatre, and inpatient ward). However, the Biological Safety Cabinet (BSC) class II, previously acquired, was placed in a normal room. Therefore, an oncology pharmacy unit was rebuilt according to USP 797 chapter. The new construction was conducted by an experienced company, thus ensuring the compliance to strict standards and procedures. The supervision of the construction of this oncology pharmacy unit, personal training and internal procedures had the support of the Portuguese Institute of Oncology—Porto. The clean room and equipment performance testing certification programme was performed by an independent international company. The new facility has now a Buffer room with negative pressure, where the BSC is located (this area is limited to the preparation and staging of components and supplies) and an anteroom (Figure 2). The training and usage of a closed system transfer device (CSTD) was also advised and then implemented.

### Sagrada Esperança Clinic

**Figure 3.**
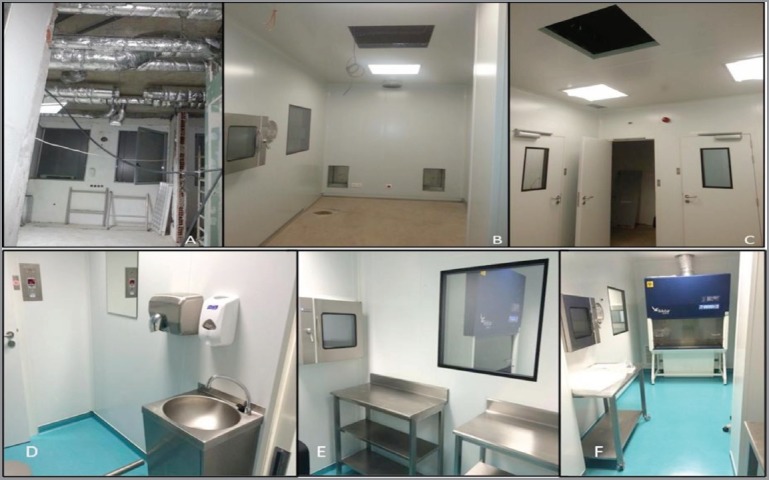
Sagrada Esperança Clinic oncology pharmacy unit before and after construction. Before: (A) Building (making of) HVAC to the buffer room. (B) Building the buffer room in modular construction. (C) Building the anteroom in modular construction. After construction meeting USP 797 criteria [36]: (D) Final anteroom. (E) Final anteroom (making ready area) connected through a pass through to the buffer room. (F) Final Buffer room with BSC type II B2.

SEC decided to set up an oncology pharmacy unit, given the increasing number of cancer patients seeking treatment each year. A dedicated building is currently under construction, where the preparation and infusion chemotherapy unit will be hosted. The location determined for the construction of the oncology pharmacy is in a central area of the hospital, allowing a quality replay to the whole hospital, without compromising the improvement of safety and control of biohazards and the discarding and ulterior destruction of waste chemotherapy (Figure 3). SEC acquired the appropriate technology resources that may also help other cancer units in Luanda, as a backup. The construction is being conducted by an experienced company. The supervision of the construction of this oncology pharmacy unit, personal training and implementation of internal guidelines has the support of Oncocirurgia Saúde e Ensino, Lda, Angola. The clean room performance testing certification programme will then be performed by an independent international company. SEC developed a HDs waste management unit, including incineration at 1100°C which will also support all cancer units in Luanda in this field.

### Angolan Institute of Cancer Control

This hospital has foreign assistance and trained technical personnel, including those preparing chemotherapy. There is a BSC, but it is located in a normal room without confinement. Professionals do use some individual safety equipment; however, the individual and environmental risk is high. On the other hand, it is urgent to reorganise the internal guidelines of HDs management.

With the construction of the two new oncology pharmacy units in Luanda (GC and SEC), which could provide a backup to preparation if necessary, making this the right moment to engineer a new oncology pharmacy unit. There is already an architectural plan for this new building, which incorporates a modern and economic solution that may include compounding aseptic containment isolators (CACI), adequate clean rooms and anterooms, according to the evidence-based international guidelines on handling HDs and related wastes.

The main option was prefabricated modular clean rooms with BSC class II B2. The costs varied between 400,000 USD and 800,000 USD, according to the amount to be constructed and the option of duplicate facilities to account for technical problems. However, a stick-built clean room will be adopted due to the future plans for building facilities.

Through the Ministry of Health, the sterile preparation of chemotherapy and waste management is currently under regulation. The main difficulties detected, in this second visit, were the lack of technical training of the pharmacy team and the affordability of HDs. Finished installations were already certified.

## Discussion

The objective of this article is to report the results of the Angolan experience in order to make general recommendations for the general planning of oncology pharmacies in low and middle income African countries.

First of all, investment in staff, equipment and facilities is required. National guidelines for a safety policy for handling HDs and related waste are also required.

### Potential solutions

When implementing an oncology pharmacy unit, a standard design model includes a centralised oncology pharmacy with an accurate, safe and efficient compounding sterile preparation unit and an oncology ambulatory pharmacy. Proper handling of HDs and microbiological control, based on good manufacturing practices (GMP) is essential. The health ministry should garner financial resources to build and maintain public compounding facilities.

Access to areas where HDs are stored and prepared should be restricted to authorised staff, avoiding risks to inexperienced personnel or unawareness.

The location of the HDs compounding area should be located away from break rooms and refreshment areas for the staff, patients or visitors, in order to reduce risk of exposure and contamination from these soiled areas. Signs designating the hazard should be prominently displayed before entry into the HDs area.

Different options of facilities may be drawn, which are as follows:

Stick-built clean rooms are complex construction projects, despite the limited design restraints imposed by this type of construction. These rooms are not limited in height or width but imply high costs, whether in time and in construction management. The availability of materials and cost feasibility must also always be considered. Pre-fabricated clean rooms are much more “modular” than traditional stick-built rooms and they can be enclosed in a previous existing space. Some constraints to modular construction are columns, ceiling height and exterior walls; nevertheless, they are a cheaper and quicker option. They are also an option for hospitals that need some flexibility. Flexibility and modularity are often used interchangeably; however, one should review these characteristics distinctly because most modular facility designs turn into a traditional facility layout, losing that flexibility. The truly modular clean room built in mobile containers is another option [27].

## Primary engineering control

### Compounding aseptic and containment isolators—option one

The HDs sterile preparation room should include compounding aseptic and containment isolators (CACI), providing an ISO class 5 within the cabinet, which must operate at a negative non-recirculating air pressure, relatively to the negative surrounding room (ISO class 7), thus limiting exposure to hazardous drug and meeting criteria of the USP 797 chapter. Isolators have become an effective option, in an effort to develop compliance strategies for compounding facilities. Traditional equipment, used to provide the critical sterile manufacturing zone (ISO Class 5), protects the product with a unidirectional flow of particle-free (HEPA filtered) air. Barrier isolators go one step further. The use of gloves and viewscreens provides a physical barrier between the operator and the product. However, users should not adopt a false sense of security with these systems [24]. Isolators are not “closed boxes” that eliminate concern for proper disinfection and aseptic technique. They are only contamination control equipments reducing the possibility of contamination but do not replace safe work practice related to aseptic technique and protection.

### Biological safety cabinet—option two

Class II, type B2, biological safety cabinets with total exhaustion and protective glass, should be used and located in a buffer area with negative pressure, meeting the standards set in the USP 797 chapter. This type exhausts all inflow and downflow air without recirculation inside the cabinet or return to the clean room. These cabinets may be used with volatile toxic chemicals and radionucleotides. Environmental protection is provided when the cabinet exhaust air is passed through a HEPA/ULPA filter [28]. The sterile preparation room should ideally have a minimum surface area of 10 m^2^.

### New technologies—option three

This includes robotic automation, which can compound sterile doses of hazardous drugs. These robots reduce the occupational exposure of health care workers during the compounding process, yet like the other options, are not perfect, most of all because they require human staff to load and clean and most of the time they do not give a quick performance. They are generally more expensive and do not substantially reduce serious medication errors [29, 30].

Other important concepts

1. A pass through equipped with sealed doors allows the transfer of drugs and equipment between an anteroom area (making ready area) and the preparation cleanroom (buffer room).
2. Negative pressure gradients between the buffer area and the other adjacent rooms in the pharmacy are required, as well as segregation, in order to avoid spreading contaminants. On the other hand, the negative pressure should not be so strong that can cause environmental, microbial or particulate contamination into the cleanroom.
3. Emergency access to water for the removal of hazardous drugs from eyes and skin should be available close by.
4. The pharmacy should also include an unpacking room/area, a segregated storage room/area for hazardous, a storage area for cleaning equipment and a data entry room/area.
5. Total exhaustion (and vented to the outside through filtration) should be always considered when choosing a PEC.
6. A maintenance plan and repair should always be defined and applied.

A common misconception surrounding CACIs is that using one will automatically guarantee USP 797 chapter compliance. However, a designated space with restricted access and negative pressure is also required. Furthermore, it is a misconception that sterile glove changes occur less frequently with CACIs. CACI gloves must be maintained and changed regularly. In fact, they need to be changed just as often as with BSCs. In CACI training and in SOPs, the location of clean or first air within the unit should be stressed. Since these devices are unidirectional (top to bottom), the positioning of the product in the unit is important, as is hand position and aseptic technique [31]. The misunderstanding also happens with personal protective equipment (PPE) of operators and buffer room with negative pressure, which is also a requirement.

The comparison of design and operating costs between controlled atmosphere areas (CAA) with isolators and CAA with laminar flow biological safety cabinets (BSC), in five European countries, revealed that preparation costs in group BSC appears higher than in the isolators group [32]. However, this study includes preparation of cytotoxic drugs, total parenteral nutrition (TPN) and others. As it applies not only for preparation of chemotherapy, it is difficult to overextend the data to this type of handling in particular.

## HD-related waste

HDs waste includes any residual hazardous drug, the materials, or equipment associated with the preparation, transport, and administration of the drug therapy. It includes unused cytotoxic pharmaceuticals, contaminated waste from preparation processes, sharps and syringes, ampoules and vials, intravenous infusion sets and containers, drug bottles and packaging that have been in contact with HDs, drug administration devices, contaminated personal protective equipment, materials used to clean HDs contaminated equipment or spills, contaminated body substance receptacles and contaminated specimens from the laboratory [33]. HDs waste should be managed separately from other types of special waste and from other wastes generated in a clinical setting and that are not assessed or classified as HDs waste. The crucial steps for waste management include containment, segregation, storage, disposal and treatment. Incineration (at 1100°C) is the only approved technology for treating HDs waste. All incinerators used for the treatment of cytotoxic waste must be licensed by the local and international environment authorities [10, 34].

## National regulations necessities

Several years of data on adverse effects of exposure to hazardous drugs, including in Africa, have sadly not been sufficient to raise enough awareness, and the risks remain a reality. The absence of legislation in several countries is the proof of it. Nevertheless, South African authorities and scientific associations developed guidelines and recommendations. Still, tremendous efforts should be conducted by all African nations in order to ensure safety at all steps of chemotherapy preparation, infusion and management of waste [35].

## Conclusions

HDs should be handled under conditions that can promote patient and worker safety, environmental protection and infection prevention. Compounding of HDs requires appropriate administrative controls, PPE, work practices, engineering, and environmental controls. Sterile HDs compounding should be performed in a PEC which can provide an ISO Class 5 critical area and must be physically located in a buffer area, meeting USP 797 chapter compliance. Emerging regulations, more investment in personal training, updating of the facilities, in order to comply to state of the art standards, are crucial tasks to be taken into account in low- and middle-income African countries.

## Conflicts of interest

Authors declare no conflicts of interest.

## Authors’ contributions

This study is conceptualised and designed by Lúcio Lara Santos. Acquisition, analysis, and interpretation of data are done by Ana Vaz Conceição, Dora Bernardo, and Lúcio Lara Santos. Dora Bernardo, Ana Vaz Conceição, Lygia Vieira Lopes, Fernando Miguel, Fernando Monteiro, Fernanda Bessa, Cristina Santos, João Blasques Oliveira, and Lúcio Lara Santos drafted or revised the article for important intellectual content. All authors read and agreed to the final version of this manuscript. Ana Vaz Conceição, Dora Bernardo, and Lygia Vieira Lopes equally contributed to this study.

## Acknowledgments

We thank Alexandre Santos and Mara Rodrigues for the correction of the article in English.

## Figures and Tables

**Table 1.** Oncology pharmacy—risk management and training: a diagnosis of the situation in 2012.

Oncology pharmacy, risk management, and training: a diagnosis of the situation (2012)^1^			
Questions presented^*^:	IACC	CG	CSE
Are there certified physical facilities and equipment for drug preparation (centralised unit) and infusion, surgical, and radiation treatments?	No	No	No
Is there a primary engineering control (PEC)?	Partially	Partially	No
Is there technical equipment for drug preparation?	Partially	No	No
Is there technical equipment for drug infusion?	Yes	No	No
Is there a written procedure for acquisition, preparation, and administration of cytotoxic drugs?	Partially	Partially	Partially
Is there a quality assurance in all areas, risk management, and safety requirements?	Partially	Partially	Yes
Are there protocols for prescription and administration of cytotoxic drugs?	Yes	Partially	Partially
Is there an adequate hazardous storage?	No	No	No
A periodical policy review and control of toxic waste is being performed?	No	No	Partially
Is there participation in oncology teaching and clinical research?	Yes	Yes	Yes
Does the institution screen the training needs to define an educational programme?	Yes	Yes	Yes

1 According to the cancer units assessment checklist for low- or middle-income African countries [21]

* Questions should be answered with Yes, Partially, No or Not Applicable

**Table 2.** Oncology pharmacy, risk management, and training: action plan (2012).

Oncology pharmacy, risk management, and training: action plan (2012)			
Questions presented:	IACC	CG	CSE
Are there certified physical facilities and equipment for drug preparation (centralised unit) and infusion, surgical, and radiation treatments?	A segregated and compounding area must be built	Improvement of a segregated and compounding area must be performed	A segregated and compounding area must be built
Is there a primary engineering control (PEC)?	Equipment must be acquired	No action needed	Equipment must be acquired
Is there technical equipment for drug preparation?	Equipment must be acquired	Equipment must be acquired	Equipment must be acquired
Is there technical equipment for drug infusion?	Equipment must be acquired	Equipment must be acquired	Equipment must be acquired
Is there a written procedure for acquisition, preparation and administration of cytotoxic drugs?	A standard operation procedure must be written	A standard operation procedure must be written	A standard operation procedure must be written
Is there a quality assurance in all areas, risk management and safety requirements?	A standard operation procedure must be written	A standard operation procedure must be written	A standard operation procedure must be written
Are there protocols for prescription and administration of cytotoxic drugs?	Improvement of protocols must be performed	Must be rewritten	Must be rewritten
Is there an adequate hazardous storage?	A segregated area must be built	A segregated area must be built	A segregated area must be built
Is a periodical policy review and control of toxic waste being performed?	SOP must be written and contaminated waste destroyed within international rules	SOP must be written and contaminated waste destroyed within international rules	SOP must be written and contaminated waste destroyed within international rules
Is there participation in oncology teaching and clinical research?	Yes	Yes	Yes
Does the institution screen the training needs to define an educational programme?	Training of the pharmacy team must be performed	Training of the pharmacy team must be performed	Training of the pharmacy team must be performed
